# ALZHEIMER’S DISEASE AND RELATED DEMENTIAS IN MEXICAN AMERICAN MEDICARE BENEFICIARIES

**DOI:** 10.1093/geroni/igz038.435

**Published:** 2019-11-08

**Authors:** Soham Al Snih, Lin-Na Chou, Brian Downer, Mukaila Raji, Yong-fang Kuo, Kyriakos Markides, Kenneth Ottenbacher

**Affiliations:** 1 University of Texas Medical Branch at Galveston, Galveston, Texas, United States; 2 The University of Texas Medical Branch, Galveston, Texas, United States; 3 University of Texas Medical Branch, Galveston, TX, Galveston, Texas, United States

## Abstract

Objective: To determine the prevalence and incidence of Alzheimer’s Disease and Related Dementias (ADRD), and to identify the socio-demographic and health characteristics of Mexican-American older adults with ADRD. Methods: Data are from wave 5 (2004/05) of the Hispanic Established Population for the Epidemiological Study of the Elderly linked with Centers for Medicare and Medicaid Services files. We studied 1166 participants of which 927 did not have an ADRD diagnosis before wave 5 interview and followed until 2016. Measures included socio-demographics, medical conditions, depression, physical function, Mini-Mental-State- Examination (MMSE), body mass index (BMI), disability, and ICD-9-CM codes for ADRD. Results: A total of 424 participants had an index diagnosis of ADRD during 11-years. The total prevalence rate ranged from 31.6% in 2006 to 72.8% in 2016, and the total incidence rate ranged from 9.3% in 2006 to 15.8% in 2016. The prevalence rate ranged from 30.3% to 69.7% in men and 32.5% to 74.1% in women. The incidence rate ranged from 8.5% to 12.9% in men and 9.8% to 12.9% in women. Those with ADRD were significantly more likely to be older (82.1 versus 81.6 years; p-value=0.024) and to have a lower score in the MMSE (21.1 versus 21.7; p-value=0.013) compared with whole sample (N=927). Non-significant differences were observed by sex, education, medical conditions, BMI, depression, physical function or disability compared with whole sample. Conclusions: The prevalence and incidence rates of ADRD in Mexican-American Beneficiaries is high. These findings underscore the need for clinical services and caregiving resources in this population.

